# Editorial: Advances in gut microbiota, metabolites and the metabolic disorders

**DOI:** 10.3389/fmolb.2026.1937318

**Published:** 2026-08-03

**Authors:** Hui Han, Jing Wang, Martine Schroyen, Bao Yi

**Affiliations:** 1 State Key Laboratory of Animal Nutrition and Feeding, Institute of Animal Science, Chinese Academy of Agricultural Sciences, Beijing, China; 2 College of Animal Science and Technology, Hunan Agricultural University, Changsha, China; 3 Precision Livestock and Nutrition Unit, Gembloux Agro-Bio Tech, University of Liège, Gembloux, Belgium

**Keywords:** gut microbiota, intervention, metabolic disorders, metabolites, obesity

The growing recognition of the gut microbiota and its associated metabolites as central regulators of host physiology has reshaped our understanding of metabolic disorders. The Research Topic entitled “*Advances in Gut Microbiota, Metabolites, and Metabolic Disorders*” highlights the multifaceted roles of microbial ecology and metabolite profiling in related diseases. The articles in this Research Topic explore how gut microbiota and its metabolites interact with the host to influence metabolic and immune processes. These metabolic disorders not only include the most common conditions such as obesity and cardiovascular diseases, but also involve some clinical illnesses closely related to various gastrointestinal diseases and metabolic disorders, including rosacea, sudden sensorineural hearing loss, and *Helicobacter pylori* infection.

A central theme emerging from this Research Topic is the identification of disease-associated metabolites alterations and their potential microbial origins. Zhang et al. performed untargeted blood metabolomic analyses across different subtypes of rosacea. Their findings provide important insights into how systemic metabolic alterations distinguish clinical subtypes of rosacea, suggesting that metabolic profiling may aid in disease stratification. Notably, they identified changes in metabolites potentially derived from the gut microbiota, supporting a role of the gut microbiota in inflammatory disease development. Similarly, Ma et al. conducted untargeted plasma metabolomic profiling in patients with sudden sensorineural hearing loss and identified distinct metabolic signatures associated with the condition. In particular, they reported a significant decrease in indole-3-acetamide, a tryptophan-derived metabolites linked to gut microbial metabolism. The authors proposed that the reduced level of indole-3-acetamide may contribute to disease pathogenesis through the promotion of inflammation responses. Together, these findings highlight the relevance of microbiota-related metabolic alterations in disease development.

Beyond metabolite-centered findings, this Research Topic further expands the scope to microbial exposure and infection, strengthening the link between microbiota and metabolic health. Liu et al. reported an association between *H. pylori* infection and alterations in the apolipoprotein B/apolipoprotein A1 ratio, suggesting a potential link between microbial exposure and cardiovascular risk. This finding broadens the scope of microbiota-related research beyond commensal microbial communities to include pathogen-associated metabolic consequences.

Importantly, beyond observational and associated findings, studies in this Research Topic address whether gut microbiota and their metabolic outputs can be actively modulated to influence disease development. Dong et al. demonstrate that swimming training modulates cecal microbial community and their metabolic outputs in rats fed a high-fat diet, highlighting physical activity as a modifiable factor influencing host-microbiome interactions. Complementing this, Liu et al. showed that electroacupuncture and manual acupuncture exert differential effects on gut microbiota composition and associated metabolic pathways in spontaneously hypertensive rats, providing mechanistic insights into how traditional therapeutic interventions may influence systemic metabolism. These intervention-based studies underscore the transactional potential of targeting microbiota and its metabolites.

Collectively, these studies underscore the complex network involving gut microbial community, microbial metabolites, and host responses in metabolism-related disorders. Advancing our understanding in this field may facilitate the identification of novel biomarkers and therapeutic targets. Nevertheless, several challenges remain before these findings can be translated into clinical applications. The heterogeneity of microbiome composition, the dynamic nature of metabolite production, and the difficulty in establishing causal relationships require more integrative approaches. Future studies leveraging multi-omics integration, longitudinal designs, and mechanistic validation will be essential to move from association to intervention.

In summary, this Research Topic expands our understanding of the role of gut microbiota and its metabolites across diverse disease contexts. By bridging observational findings with emerging intervention strategies, it provides a foundation for future efforts to harness microbiota-metabolite interactions in the prevention and treatment of metabolic disorders.

